# Surgical Experiences in the Present War

**Published:** 1914-12-05

**Authors:** 


### SURGICAL EXPERIENCES IN THE PRESENT WAR.

(Contributions to this Section are Invited.)

### A Fruitful Field of Research.

### GAS GANGRENE.

MEDICAL men who have attended wounded soldiers in the present campaign have been much distressed at the frequency with which gangrenous infection of wounds has occurred. Gangrenous, gas-containing abscesses have been common, while cases of septicæmia and sloughing amputation flaps are not unknown. Statistics are not available to show the exact percentage of wounds which become so infected. However, the cases are sufficiently numerous among the wounded, even in our hospitals at home, to demand serious thought upon the subject, and, as the symptoms of gas gangrene often appear soon after the injury, sometimes within thirty-six hours, it is probable that such cases are more numerous in the hospitals on the Continent than in those at home. Unhappily the causative bacilli are spore-bearing, and therefore resistant to the ordinary means of wound-disinfection; and although strong antiseptics, when brought into contact with these organisms, may so affect them as to put a limit to their pathological activities, yet these agents cannot be relied upon as effective. Apart from the conditions under which first-aid is rendered in war, the disinfection of gunshot wounds is often rendered impossible, except under anæsthesia, because they contain bullets, fragments of other projectiles, or pieces of earth-soiled clothing deeply imbedded in the tissues. Serum treatment in these circumstances seems to offer the only reasonable hope of combating the evil, and in spite of the fact that several varieties of earth-borne bacteria appear to be included amongst the evil agents of gas gangrene, there is a good prospect that the difficulty raised by this fact may be overcome, and that a potent and successful prophylactic serum may be evolved. It is with satisfaction, therefore, that we learn, from an article communicated to last week's *British Medical Journal* by Sir Anthony Bowlby and Mr. Sidney Rowland, that the Lister Institute has already been busy with a bacteriological investigation into the causes of gas gangrene with the view to the preparation of a serum for prophylactic use.

Incidentally the activities of the Lister Institute on these lines may be regarded as additional evidence, if such be needed, of the wisdom of its governors in their refusal to be absorbed by the Committee for Medical Research, established under the National Insurance Act. Institutions controlled by the Government are so much trammelled with their own inertia that they seldom achieve renown by their versatility and nimbleness of foot.

### ANTI-TETANUS SERUM.

As experience accumulates it becomes increasingly clear that prophylactic injections of anti-tetanus serum are successful to some extent, at any rate, in preventing tetanus. Whether tetanus can be avoided infallibly by this treatment remains to be proved, and no doubt the Army Medical Department will have statistics available at the end of the war sufficiently large to allow of the deduction of correct conclusions on this point. In the meantime it is to be noted that cases of tetanus have been less numerous among the wounded since the use of prophylactic serum was employed on a large scale. In one of the large emergency hospitals at home there have been, since the outbreak of the war, four cases of tetanus amongst the wounded, all of which cases had a fatal ending, and all of which occurred in men who had not received prophylactic injections of anti-tetanus serum. Of course, the significance of small figures in a matter of this kind is not very great; neither is it negligible. Tetanus is a most painful and distressing disease, and any remedy that offers a hope of combating it will be most welcome for its own sake. But it will be all the more welcome as an indication that not only the tetanus bacillus, but other earth-bred, spore-bearing, pathogenic bacteria also may be met and defeated by similar weapons, and that war may be so robbed of some at least of its worst terrors.

